# A novel therapeutic strategy for osteosarcoma using anti-GD2 ADC and EZH2 inhibitor

**DOI:** 10.1186/s40364-025-00800-3

**Published:** 2025-06-18

**Authors:** Jing Shan, Zicheng Lin, Harunor Rashid, Peng Huang, Lei Qiang, Yihao Liu, Guanlu Shen, Yuchen Li, Jiaming Cui, Zhi Su, Hanbo Wang, Bixuan Cao, Cheng Zhou, Veysel Kayser, Bo Ning

**Affiliations:** 1 School of Pharmacy, The University of Sydney, Sydney, NSW 2006 Australia; 2 National Children’s Medical Centre Department of Orthopaedics Surgery, Children’s Hospital of Fudan University, Wanyuan Road 399, Minhang District, Shanghai, 201102 China; 3 Sydney Infectious Diseases Institute, The University of Sydney, Westmead, NSW 2145 Australia; 4 Department of Orthopaedic Surgery, Children’s Hospital of Nanjing Medical University, Nanjing, Jiangsu 210000 China; 5 Shanghai Key Laboratory of Orthopedic Implant, Shanghai Jiao Tong University School of Medicine, Shanghai , 200011 China; 6 School of Pharmacy, Jiangsu Ocean University, Lianyungang, Jiangsu 222000 China; 7 Affiliated hospital 2 of Nantong university, No. 666, Shengli Road, Nantong City, Jiangsu Province China; 8 School of Exercise and Health, Shanghai University of Sport, Shanghai , 200438 China; 9 Department of Biomedical Engineering, Stony Brook University, Stony Brook, New York , 11794 US; 10 Department of Orthopedics, The Third Affiliated Hospital of Anhui Medical University, The First People’s Hospital of Hefei, 390 Huaihe Road, Hefei , Anhui 230061 China; 11 Affiliated Children’s Hospital of Anhui Medical University, Hefei, Anhui 230061 China

**Keywords:** Antibody drug conjugates, Osteosarcoma, GD2 upregulation, EHZ2-inhibitor, Tazemetostat

## Abstract

Osteosarcoma is the commonest malignant bone tumour in children, with a poor prognosis due to limited treatment options. Antibody–drug conjugates (ADCs) offer targeted therapeutic potential but are limited by the low expression of specific antigens like disialoganglioside (GD2) on osteosarcoma cells. This study aimed to enhance the efficacy of an anti-GD2 ADC by combining it with the enhancer of zeste homolog 2 (EZH2) inhibitor tazemetostat to upregulate GD2 expression and improve treatment outcomes. We constructed an ADC by conjugating naxitamab to mertansine (DM1) via an SMCC linker. Human osteosarcoma cell lines (U2OS and 143B) were treated with tazemetostat to increase GD2 expression. In vitro experiments included apoptosis assays, gene expression analysis, and flow cytometry. We evaluated in vivo efficacy in mouse xenograft models by measuring tumour growth and pulmonary metastases. Tazemetostat treatment significantly increased GD2 expression in U2OS and 143B cell lines. The anti-GD2 ADC induced apoptosis via the mitochondrial pathway, as shown by increased expression of apoptosis-related genes and higher apoptosis rates. In vivo, the ADC significantly inhibited tumour growth and reduced pulmonary metastasis. These therapeutic effects were further enhanced when the ADC was combined with tazemetostat. Combining anti-GD2 ADC therapy with EZH2 inhibition effectively improves targeted treatment for osteosarcoma. Tazemetostat upregulates GD2 expression, enhancing the ADC’s efficacy. This dual-approach strategy demonstrates the potential of integrating epigenetic modulation with targeted drug delivery, offering a promising path for improving outcomes in hard-to-treat cancers.

**Supplementary Information:**

The online version contains supplementary material available at 10.1186/s40364-025-00800-3.

## To the editor

Osteosarcoma is the most common malignant bone tumour, accounting for nearly 56% of all bone sarcomas [1]. Despite conventional treatments, osteosarcoma recurs in 30–50% of patients, with survival rates declining from ~ 68% in localised cases to just 20–30% when metastases are present. This highlights the urgent need for more advanced therapies [2, 3]. ADCs offer targeted therapeutic potential but are limited by the relatively low or heterogeneous expression of disialoganglioside GD2 on osteosarcoma cells [4, 5]. This study aimed to enhance the efficacy of an anti-GD2 ADC by combining it with an enhancer of zeste homolog 2 (EZH2) inhibitor, tazemetostat, to upregulate GD2 expression.

The detailed methodology is in Supplementary File S1. Briefly, the study comprised: (i) analysis of publicly available data from the TARGET (Therapeutically Applicable Research to Generate Effective Treatments) database; (ii) spectroscopic validation and drug-antibody ratio [6]; (iii) in vitro assessment of ADC efficacy in U2OS and 143B cell lines (ATCC, conducted in triplicate); and (iv) in vivo evaluation across three phases—monotherapy, early-phase therapy (commencing on Day 4), and late-period therapy (commencing on Day 14), each with five replicates.

**Fig. 1:**
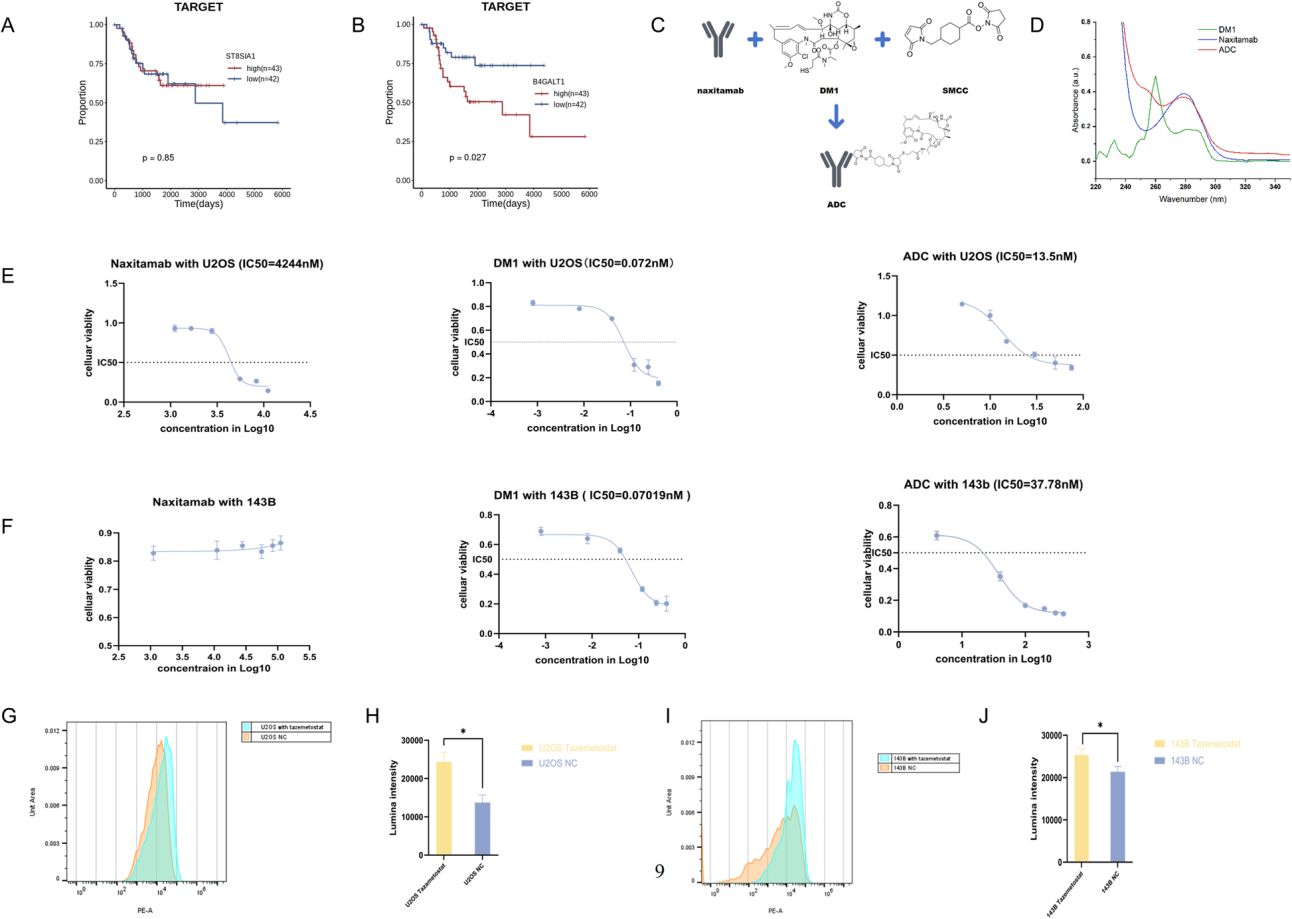
The results of experiments in vitro. **A** The *ST8SIA1* had no significance with GD2 expression. The *B4GALT1* had significance with GD2 expression. **C** Schematic representation of the anti-GD2 ADC synthesis process, involving the conjugation of naxitamab with the cytotoxic agent DM1 via SMCC linker. UV–Vis spectra show distinct absorbance patterns for DM1, naxitamab, and the ADC. The ADC spectrum combines features of both DM1 and naxitamab, confirming successful drug-antibody conjugation. (E) CCK-8 assay results showing the cytotoxic effects of free DM1, naxitamab, and ADC in U2OS cell lines; the IC50 of naxitamab: 4244 nM; the IC50 of free DM1: 0.072 nM; the IC50 of ADC: 13.5 nM. (F) CCK-8 assay results showing the cytotoxic effects of free DM1, naxitamab, and ADC in 143B cells; the IC50 of naxitamab: NA; the IC50 of free DM1: 0.07 nM; the IC50 of ADC: 37.38 nM. (G-H) Flow cytometry analysis of GD2 expression in the U2OS cells treated with tazemetostat and the control groups. Flow cytometry analysis of GD2 expression between the 143B cells treated with tazemetostat and the control groups. The significance levels were established at *p < 0.05, **p < 0.01, and ***p < 0.001

Clinical data analysis indicated that elevated *B4GALNT1* expression was more strongly correlated with poor survival outcomes than *ST8SIA1* (Fig. 1A–B), both of which encode glycosyltransferases involved in GD2 biosynthesis. Spectroscopic validation of conjugation showed two absorbance peaks at ~ 280 nm and 260 nm, representing ADC and naxitamab, and DM1. Following conjugation with DM1, drug-antibody ratio (DAR) was 3.7, indicating successful ADC conjugation (Fig. 1C-D).

As expected, in vitro analysis showed that cytotoxic activity of free DM1 was higher in both cell lines compared to ADC and naxitamab. U2OS displayed slightly higher sensitivity to ADC (IC50 = 13.5 nM) than 143B cells (IC50 = 37.38 nM). IC50 for naxitamab in U2OS cells was 4244 nM and no cytotoxic effect was found in 143B (Fig. 1E-F). GD2 expression levels significantly increased in both cell lines (Fig. 1G-H).

**Fig. 2:**
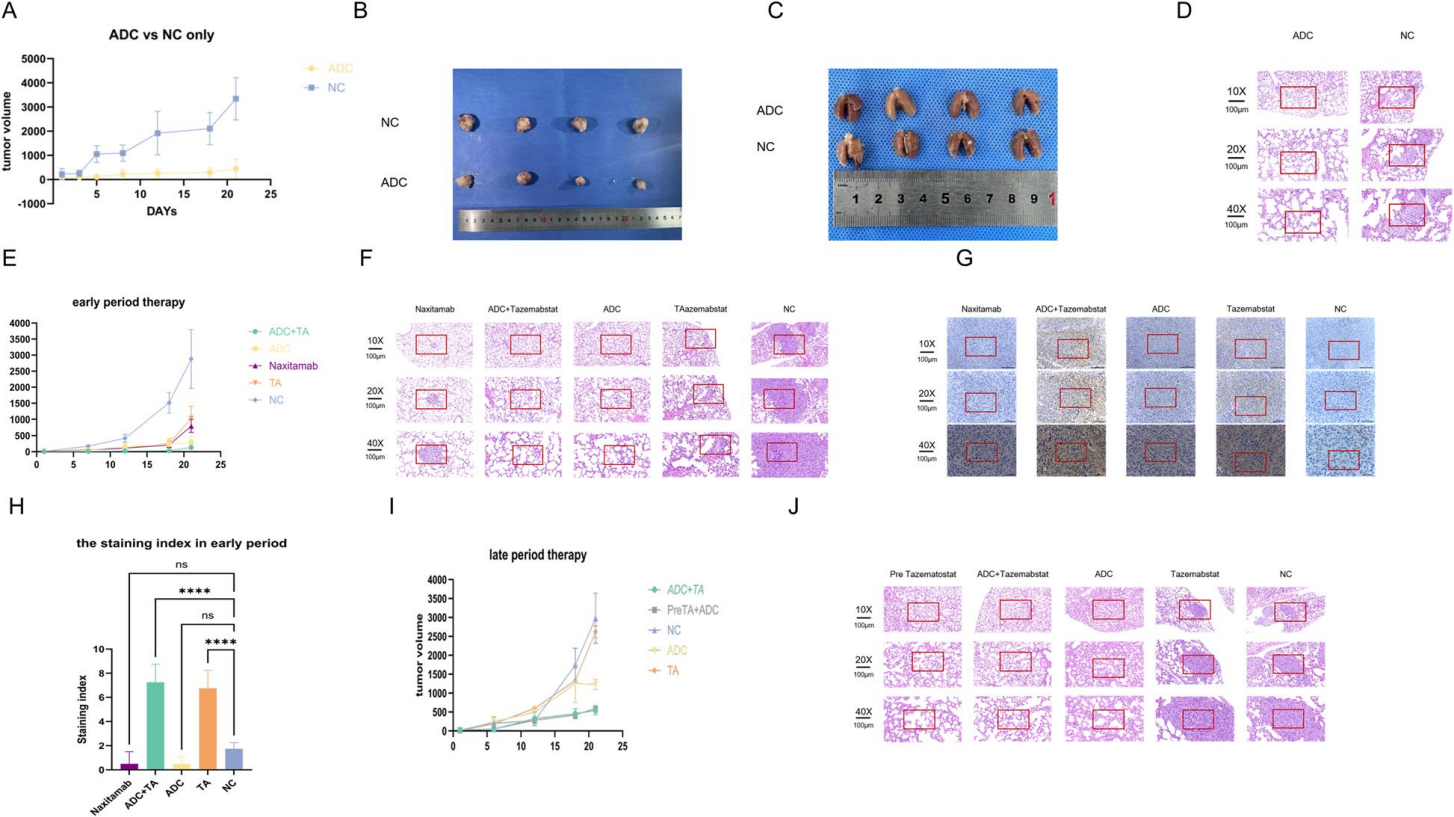
The results of experiments in vivo. **A** Tumour size changes between the mice treated with ADC and the control. Tumour volume comparison between the ADC and control groups. **C** The metastatic nodules between the ADC and control groups in the lung tissues. HE staining for pulmonary metastasis between the ADC and control groups. **E** Tumour volume comparison between the different groups for early-period therapy. **F** HE staining of lung tissues for pulmonary metastasis from the mice in different groups for early-period therapy. **G** IHC staining showing GD2 expression in the early period therapy between the different groups (the yellow is GD2 expression; the blue is negative). **H** The quantitative analysis of IHC staining of GD2 expression in the different groups for early-period therapy. Tumour volume comparison among the different groups for late-period of therapy. **J** HE staining of lung tissues for pulmonary metastasis from the mice in different groups late-period of therapy. **K** IHC staining showing GD2 expression in the late-period therapy between the different groups (the yellow is GD2 expression; the blue is negative). The quantitative analysis of IHC staining of GD2 expression in the different groups late-period of therapy. The significance levels were established at *p < 0.05, **p < 0.01, and ***p < 0.001

In vivo experiments showed tumour volume in control group was ~ 6.75 times that in treatment group (Fig. 2A-B). Haematoxylin and eosin (HE) staining of pulmonary tissues exhibited classical signs of metastasis in control group while no obvious metastasis was observed in the treatment group (Fig. 2C-D).

In early-phase therapy, the anti-tumour activity of ADC combined with tazemetostat demonstrated superior tumour growth inhibition compared to naxitamab, tazemetostat alone, and the control group (Fig. 2E), with HE staining indicating the most pronounced anti-metastatic effect (Fig. 2F), while immunohistochemistry (IHC) confirmed tazemetostat-induced GD2 upregulation in both the combination and monotherapy groups (Fig. 2G-H).

Anti-GD2 ADC with tazemetostat for late-period therapy showed robust tumour growth inhibition in the groups treated with ADC combined with tazemetostat and in the group pretreated with tazemetostat then given ADC. Moderate inhibition was observed in group receiving ADC alone, and no significant inhibition was noted in tazemetostat-alone group (Fig. 2I). The pulmonary flux values in the groups treated with ADC, ADC combined with tazemetostat, and ADC with tazemetostat pretreatment were lower than those in the tazemetostat and control groups. HE staining showed the lowest metastasis level in the three groups that received ADC (Fig. 2J). IHC results showed the highest GD2 expression in the ADC combined with tazemetostat, ADC with tazemetostat pretreatment, and tazemetostat alone groups (Fig. 2K-L), indicating that the anti-tumour effect of ADC can be enhanced with tazemetostat, even in late-stage, and that these regimens can be co-administered.

This study provides compelling evidence combinational therapy with ADC and tazemetostat is effective against osteosarcoma, and demonstrates tazemetostat upregulates GD2 expression in osteosarcoma cell lines [7, 8]. Inhibiting EZH2 has been associated with modulation in differentiation programmes, including ganglioside expression [9, 10]. The constructed anti-GD2 ADC, naxitamab-DM1, has shown significant anti-tumour activity against osteosarcoma cells likely through caspase activation [11].

In vivo experiments confirmed the therapeutic potential of combining the ADC with tazemetostat in treating osteosarcoma, even during late-period therapy. Early treatment likely benefits from a lower tumour burden, more effective targeting, and apoptosis without adaptive resistance [12].

Despite modest GD2 upregulation in U2OS and 143B cells, and the inherent limitations of the xenograft model, our findings support further investigation of this combinatorial approach, with ongoing studies aimed at elucidating the underlying mechanisms.

## Supplementary Information

Supplementary Material 1

## Data Availability

No datasets were generated or analysed during the current study.

## Abbreviations

ADC: Antibody-Drug Conjugate
CCK-8: Cell Counting Kit-8
CCLE: Cancer Cell Line Encyclopedia
DAR: Drug-to-Antibody Ratio
DM1: Derivative of Maytansine
DMEM: Dulbecco's Modified Eagle Medium
EZH2: Enhancer of Zeste Homolog 2
GD2: Disialoganglioside
HE: Hematoxylin and Eosin
IHC: Immunohistochemistry
IC50: Half Maximal Inhibitory Concentration
mAb: Monoclonal Antibody
NC: Negative control
NA: Not available
nM: Nanomolar
SMCC: Succinimidyl-Trans-4-(N-Maleimidylmethyl) Cyclohexane-1-Carboxylate
UV-Vis: Ultraviolet-Visible Spectroscopy
U2OS: Human Osteosarcoma Cell Line
