# Supplementary material for: A novel therapeutic strategy for osteosarcoma using anti-GD2 ADC and EZH2 inhibitor
Source: Biomark Res. 2025 Jun 18;13:87. doi: 10.1186/s40364-025-00800-3 (PMC12177979; doi:10.1186/s40364-025-00800-3)
Supplement: Supplementary file 1 — Supplementary Material 1 [file 40364_2025_800_MOESM1_ESM.docx]

**Supplemental File**

**Materials**

The key reagents used in this study are: humanized anti-GD2 monoclonal antibody, naxitamab (Cat. No. HY-P99206, MedChemExpress, Shanghai, China), SMCC-DM1(Cat. No. HY-101070, Cayman, NJ, USA). Amicon 50kDa cut-off filter (Cat. No. UFC805096, Darmstadt, Germany), and Dulbecco's modified eagle medium (DMEM), high glucose, with GlutaMAX (Cat. No. 10566016, Thermo, Waltham, MA, USA). Other reagents are: Mertansine (Cat. No. 22483, Cayman, Ann Arbor, MI, USA), McCoys 5A (Cat. No. 30-2007, ATCC, Manassas, VA, USA), Fetal Bovine Serum (FBS) (Cat. No. 10099141, Thermo, Waltham, MA, USA) and tazemetostat (Cat. No. T1788, TargetMol, Boston, MA, USA).

Other reagents include: Cell Counting Kit-8 (CCK-8) Cell Proliferation Assay Kit (Cat. No. B2001, Huaxia, Shanghai, China), PE anti-human Ganglioside GD2 (Cat. No. 357304, BioLegend, San Diego, CA, USA), and Ganglioside GD2 Antibody (Cat. No. sc-53831, Santa Cruz, Dallas, TX, USA).

**Cell Culture**

The U2OS cells (Cat. No. HTB-96) were obtained from ATCC (Manassas, VA, USA), while the 143B cells were generously donated by Professor Jinwu Wang from Shanghai Jiaotong University. U2OS cells and 143B cells were cultured in Corning^®^ ultra-low attachment U-flasks with vent caps canted neck T75 cm² cell culture flasks. U2OS cells were grown in McCoy's 5A medium, and 143B cells were cultured in DMEM with high glucose, both supplemented with 10% (v/v) fetal bovine serum (FBS) and 1% (v/v) penicillin/streptomycin (100 U/mL). All cultures were incubated in a humidified atmosphere at 37°C with 5% CO_2_. For washing and detaching the cells, sterile, filtered 1× PBS (pH 7.4) and pre-warmed 0.05% trypsin-EDTA were used, respectively. The cells were centrifuged at 0.3 relative centrifugal force for 5 minutes to pellet them.

**Data Source and Survival Analysis**

Data were sourced from the TARGET (The Therapeutically Applicable Research to Generate Effective Treatments) database. The transcriptome and corresponding clinical data for osteosarcoma were downloaded. Expression levels of the target gene were extracted and merged with survival data and survival status information. Survival analysis was conducted using R software version 4.1.3 (R Foundation for Statistical Computing, Austria) utilizing the “survival” and “survminer” packages to perform Kaplan-Meier (KM) survival analysis and plot the KM curves.

**Conjugate Synthesis and Characterization of Anti-GD2 ADC**

Naxitamab in formulation buffer was exchanged with PBS (0.01M, 30mM sodium bicarbonate, pH 8) to reach a final concentration of 3 mg/ml. SMCC-DM1 was dissolved in DMSO to a concentration of 1 mM and added to naxitamab at a ratio of 8:1 (SMCC-DM1: naxitamab). The mixture was allowed to react at 4°C for 4 hours. To remove unreacted SMCC-DM1, a 50 kDa cut-off filter was employed. The final product was stored in PBS (pH 7.4). FTIR spectroscopy was then performed to verify successful ADC synthesis by analyzing the presence of characteristic functional groups.

**DAR and UV-Vis spectroscopy**

UV-Vis absorbance spectra were measured over a wavelength range of 200-400 nm using a UH5300 UV-Vis spectrophotometer (HITACHI, Japan). The data were normalized and plotted with Origin (version 2021). Concentrations and DAR were determined and calculated using the Beer-Lambert law (DAR = (εAb at 260 nm - H × εAb at 280 nm) / (H × εD at 280 nm - εD at 260 nm).

**In vitro Cell Cytotoxic Assay**

U2OS and 143B cells were seeded at 5x10^3^ cells per well in 96-well plates and incubated at 37°C for 24 hours in McCoy's 5A/DMEM media with 10% FBS, respectively. Fresh McCoy's 5A/DMEM media containing 1% penicillin/streptomycin, along with free DM1, ADC, or naxitamab at the appropriate concentrations, were then added to the wells in triplicates. McCoy's 5A/DMEM media alone was used for negative control samples. Cell cytotoxicity was assessed at 72 hours using a CCK-8 assay, measured with a microplate reader (BioTek) at a wavelength of 450 nm, following the manufacturer's instructions.

**In vitro GD2 expression analysis**

Cell lines were trypsinized into suspension and washed twice with PBS + 2% FBS

before staining with PE anti-human Ganglioside GD2. Then, cells were incubated with PE anti-human Ganglioside GD2 for 15-30 min at room temperature. All the cells were filtered through a 40-μM filter and immediately measured on a Fortessa flow cytometer. The data were calculated and plotted via Flow J version 10.1.8. Triplicates have been performed in each group.

**Cell-derived xenograft tumor model experiments**

BALB/c nude mice (female, 4 weeks old, 20g) were obtained from the Shanghai Shengchang Animal Research Centre (Shanghai, China) and housed under specific pathogen-free (SPF) conditions. Following anaesthesia, 1×10^6 143B cells suspended in 50 μL PBS were orthotopically injected into the proximal tibial region of the mice. The ADC/naxitamab was given every three days as 5mg/kg or 10mg/kg via intraperitoneal injection, respectively, and tazemetostat was administrated via oral gavage as 500mg/kg twice daily. The vehicle controls for both ADC and tazemetosatat were included. Tumor volume and body weight were monitored every three to four days using calipers. Tumor volume was calculated using the formula: volume = *1/2 × length × width^2^*. The experiment was terminated if the mice showed significantly reduced activity, a body weight loss exceeding 20%, or severe cachexia, or either length or width more than 25mm.

After four weeks, the mice were euthanized, and their lungs and tumors were harvested, fixed in formaldehyde, and subsequently embedded in paraffin. Hematoxylin and eosin (H.E) staining was performed on pulmonary sections for histopathological examination. Anti-GD2 antibody staining was conducted on tumor sections to analyze GD2 expression.

**Immunohistochemistry (IHC) staining in vivo**

Both human and animal tumor samples were fixed in 4% polyoxymethylene for a minimum of 48 hours. After fixation, samples underwent gradient ethanol dehydration, were embedded in paraffin, and sectioned into 4-μm-thick slices. IHC staining was conducted to localize the expression of target GD2, using Ganglioside GD2 Antibody (Cat. No. sc-53831) at a dilution of 1:5000. Detection was performed with the GTVision™ III Detection System/Mo&Rb kit (GENE, GK500710), adhering to the manufacturer’s instructions.

**IHC staining semi-quantitation**

Immunohistochemistry staining was semi-quantitatively evaluated based on the staining index (SI) method, with positive cell proportion divided into five groups: no positive cells recorded as 0 points; <10% positive cells recorded as 1 point; 10-35% positive cells recorded as 2 points; 35-75% positive cells recorded as 3 points; >75% positive cells recorded as 4 points(1). The staining intensity (SI) was also divided into four groups: no staining recorded as 1 point; weak staining recorded as 2 points; moderate staining recorded as 3 points; strong staining recorded as 4 points. The total score was obtained by multiplying PP and SI.

**Hematoxylin and Eosin (HE) Staining in vivo**

Osteosarcoma tumor tissues were processed as follows: Paraffin-embedded sections were deparaffinized in xylene for 20 minutes, then rehydrated through a graded series of ethanol concentrations (100%, 90%, 75%) before rinsing in tap water. Frozen sections were equilibrated to room temperature for 5-10 minutes and fixed if needed before rinsing. Sections were stained with hematoxylin for 3-5 minutes, rinsed, differentiated in hematoxylin differentiation solution (Cat. No. G1039, Servicebio, China) for 2-5 seconds, and rinsed again. Bluing was performed using hematoxylin bluing solution (Cat. No. G1040, Servicebio, China) for 2-5 seconds, followed by eosin staining for 5 minutes after dehydration through ethanol. Finally, sections were cleared in xylene, mounted with neutral balsam, and covered with a coverslip. The results were examined using a pathological tissue section scanner (Pannoramic MIDI II, 3DHIETECH®, Hungary).

**Statistical analysis**

All data were summarized and presented as mean ± standard deviation (SD). Multiple comparisons were analyzed using one-way ANOVA followed by Tukey's post-hoc test. Statistical analyses were conducted with GraphPad Prism version 9.5.1 (GraphPad Software, USA). The significance levels were established at *p < 0.05, **p < 0.01, and ***p < 0.001, representing progressively increasing levels of statistical significance.

**Reference**

1. Yu AL, Gilman AL, Ozkaynak MF, London WB, Kreissman SG, Chen HX, et al. Anti-GD2 antibody with GM-CSF, interleukin-2, and isotretinoin for neuroblastoma. N Engl J Med. 2010;363(14):1324-34.
